# MiR-297 inhibits tumour progression of liver cancer by targeting PTBP3

**DOI:** 10.1038/s41419-023-06097-0

**Published:** 2023-08-26

**Authors:** Na Lu, Jiali Min, Lin Peng, Shengjian Huang, Xiahua Chai, Susu Wang, Jian Wang

**Affiliations:** 1 The Institute of Reproduction and Stem Cell Engineering, School of Basic Medical Sciences, Central South University, Changsha, China; 2 Reproductive and Genetic Hospital of CITIC-Xiangya, Changsha, China; 3 Changsha Hospital for Maternal and Child Health Care of Hunan Normal University, Changsha, China; 4 Hunan Guangxiu Hi-tech Life Technology Co., Ltd., Changsha, China; 5 National Engineering and Research Center of Human Stem Cells, Changsha, China

**Keywords:** Liver cancer, Multipotent stem cells

## Abstract

Whereas increasing evidences demonstrate that miR-297 contributes to the tumour development and progression, the role of miR-297 and its underlying molecular mechanisms in hepatocellular carcinoma (HCC) was still unclear. Here, we reported that the expression of miR-297 increased significantly in hepG2 cells after the treatment of the conditioned medium of human amniotic epithelial cells(hAECs) which can inhibit the proliferation and migration of hepG2. And the overexpression of miR-297 inhibits the cell proliferation, migration and invasion of HCC cell lines in vitro and suppressed the tumorigenesis of HCC in vivo. Polypyrimidine tract-binding protein 3 (PTBP3) was identified as a direct target gene of miR-297 in HCC cell lines, and mediated the function of miR-297 in HCC cells. In clinical samples, miR-297 levels have a tendency to decrease, but there are no statistically significant differences. Furthermore, in vitro cell experiments confirmed that overexpression of miR-297 could inhibit the PI3K/AKT signaling pathway by down-regulating PTBP3 expression, thereby inhibiting the proliferation, migration and invasion of HCC cells. In conclusion, our results revealed that miR-297 could down-regulate the expression of PTBP3 and inhibit the activation of PI3K/AKT signaling pathway, thereby preventing HCC growth, migration and invasion.

## Background

Liver cancer is the sixth most common malignancy and the fourth most frequent cause of cancer-related death worldwide, accounting for approximately 50% of the total number of cases and deaths annually in China alone [1, 2]. Hepatocellular carcinoma (HCC) comprises 75%-85% of primary liver cancer cases, which is characterized with poor prognosis and high mortality [3]. Despite recent advances in surgical techniques and improvements in the 5-year survival rate, the long-term prognosis for HCC patients remains poor due to the high rate of intrahepatic recurrence and metastasis [2–4]. Thus, it is urgent to clarify the molecular mechanisms underlying the tumorigenesis and progression of HCC for better treatment. Human amniotic epithelial cells (hAECs), a kind of human placental adult stem cells derived from fetal membranes, are a novel potential cell source for cellular therapy and tissue regeneration [5]. A growing body of evidence has demonstrated the anti-tumour properties of hAECs [6, 7]. In our study, we demonstrated that the treatment of the conditioned medium of human amniotic epithelial cells(hAECs) which can inhibit the proliferation and migration of hepG2. However, the molecular mechanisms underlying the anti-tumour effects of hAECs remain unclear.

To determine the mechanism of growth inhibition, we performed miRNA array to screen the differential expression of miRNAs. We demonstrated that the expression of miR-297 increased significantly in HepG2 cells after treatment with conditioned medium from human amniotic epithelial cells (hAECs). MicroRNAs (miRNAs) are a group of endogenous evolutionarily conserved single-stranded noncoding small RNAs of 18-25 nucleotides that mediate mRNA degradation or translational repression by binding to complementary sequences within the 3′-untranslated regions (UTR) of target mRNA [8, 9]. MiRNAs are widely involved in the process of cancer initiation, development and progression, such as cell proliferation, apoptosis, metastasis and drug resistance, as posttranscriptional regulators of gene expression, thus suppressing or promoting the development of cancers [10–12]. Recently, miR-297 was found to be involved in tumour development [13–16]. Xu et al. [13] revealed that overexpression of miR‐297 modulates multidrug resistance in human colorectal carcinoma by downregulating MRP-2. Sun et al. [14] showed that miR-297 acts as an oncogene by targeting GPC5 in lung adenocarcinoma. Kefas et al. [15] demonstrated that miR-297 is a novel and physiological regulator of glioblastoma survival by targeting DGK-α. Moreover, studies have shown that the expression of miR-297 decreased significantly in HCC tissues compared with normal liver tissues [16]. However, the role of miR-297 and its underlying mechanisms in liver cancer are still unclear. As the tumour-suppressive role for miR-297 in liver cancer is in contrast to its known tumour-promoting role in other types of cancer such as colorectal cancer, lung adenocarcinoma, and glioblastoma, we further investigated the effect of miR-297 on the tumour progression of liver cancer.

In this study, we demonstrated that the expression of miR-297 increased significantly in HepG2 cells after treatment with conditioned medium from human amniotic epithelial cells (hAECs), which can inhibit the proliferation and migration of HepG2 cells. The overexpression of miR-297 inhibits the proliferation, migration and invasion of HCC cells. Polypyrimidine tract-binding protein 3 (PTBP3) was identified as a direct target gene of miR-297 and mediated the function of miR-297 in HCC cells. The results indicated that miR-297 plays an important role in the development of HCC and acts as a potential therapeutic target for HCC.

## Methods

### Cell culture

The HepG2, Huh7, and HEK293T cell lines and hAECs were obtained from the National Engineering Research Center of Human Stem Cells of Central South University, Changsha, China. The HCC cell lines and HEK293T cells were cultured in Dulbecco’s modified Eagle’s medium (DMEM, Gibco, USA) supplemented with 10% foetal bovine serum (FBS, Gibco, USA) at 37 °C in a 5% humidified environment of CO_2_. hAECs were cultured in Dulbecco’s modified Eagle’s medium (DMEM, Gibco, USA) supplemented with 10% foetal bovine serum (FBS, Gibco, USA) and EGF (10 ng/ml, R&D, USA). The medium of HepG2, Huh7, and HEK293T cell lines was changed on alternate days. The culture medium of hAECs was changed every 3 days, and the cells were harvested for further experiments when 80% confluence was reached.

### Preparation of conditioned medium

hAECs were cultured in 10 mm petri dishes at 37 °C, and the original culture system was abandoned when 80% confluence was reached. Then, the cells were washed three times with PBS, the fresh culture system (without growth factor) was replaced, and the conditioned medium was collected after incubation for 48 h. After centrifugation at 1500 rpm for 5 min, the supernatant was collected for further testing or stored at −80 °C.

### Lentivirus production and infection

Lentiviral particles carrying the hsa-miR-297 precursor and the flanking control sequencer were constructed by GeneChem (Shanghai, China). HepG2 cells were infected with lentiviral vector, and polyclonal cells with green fluorescent labels were selected for further experiments with puromycin (5 μg/ml; Sigma) to generate stable clones. Hsa-miR-297 expression was confirmed by RT-qPCR.

### Cell transfection

The miR-297 mimics, miR-297 inhibitor, negative control miR-NC and siRNA for PTBP3 were designed and synthesized by RiboBio Inc. (Guangzhou, China). PI3K inhibitor Ly294002 was purchased from Sigma. The sequences are listed in Supplementary Table 1. Twenty-four hours before transfection, liver cancer cell lines were plated onto a 6-well plate (Corning, China) at 30–50% confluence. The cells were not transfected with any sequence served as a blank control (blank group); other cells were transfected with 50 nM miR-297 mimics, miR-NC using Lipofectamine® 2000 reagent (Invitrogen, USA) according to the manufacturer’s protocol. And the transfection concentration of inhibitor and negative control In-NC was 100 nM. Cells were collected after 48–72 h for further experiments.

### RT-qPCR

Total RNA was extracted from liver cancer cell lines by TRIzol reagent (Invitrogen, USA) and reverse transcribed into cDNA using the GoScript™ Reverse Transcription System (Promega, USA). RT-qPCR was then performed using LightCycler® 480 SYBR Green I Master Mix (Roche, USA). For mature miRNAs, cDNA was reverse-transcribed using the Mir-X™ miRNA First-Strand Synthesis Kit (Takara, Japan) and then amplified by LightCycler® 480 SYBR Green I Master Mix (Roche, USA). U6 and GAPDH were used as internal references for miR-297 and mRNAs, respectively. The 2^−△△CT^ method was used to determine relative gene expression. The specific primers for PCR amplification are provided in Supplementary Table 2.

### Western blotting analysis

Total protein was extracted from each group of tissues and cells using ice-cold RIPA buffer (Beyotime, Shanghai, China). Antibodies included anti-AKT(Cat. No. 60203-2-Ig, Proteintech), p-AKT(Cat. No. ab38513, Abcam), PI3K (Cat. No. ab227204, Abcam), p-PI3K (Cat. No. ab182651, Abcam), E-cadherin (Cat. No. 20874-1-AP, Proteintech), N-cadherin (Cat. No. 66219-1-Ig, Proteintech), Vimentin (Cat. No. 10366-1-AP, Proteintech), PTBP3(Cat. No. bs-21038R, Bioss) and GAPDH (Cat. No. 10494-1-AP, Proteintech). The proteins were separated using 12% SDS-PAGE and then electrophoretically transferred onto a polyvinylidene fluoride membrane (Merck Millipore, USA). The membranes were blocked with 5% skim milk in TBST buffer (pH 7.5; 100 mM NaCl, 50 mM Tris and 0.1% Tween-20) for 2 h at room temperature, incubated at 4 °C overnight with primary antibodies, and then incubated with appropriate secondary antibodies (Proteintech, USA) for 2 h at room temperature. Protein bands were visualized by ECL and analysed by the Bio-Rad ChemiDoc^TM^ MP system (Bio-Rad, USA).

### Luciferase reporter assay

PTBP3 was predicted to be directly regulated by miR-297 using TargetScan software. For reporter assays, 293 T cells were cotransfected with the GV272(SV40-Luc-MCS) firefly luciferase reporter (PTBP3 WT/PTBP3 MUT) and hsa-miR-297 mimics or negative control miR-NC by Lipofectamine® 2000 reagent in 24-well plates. Luciferase activity was measured at 48 h after transfection using the Dual-Luciferase Reporter Assay System (Promega, Madison, WI, USA). Firefly luciferase activity was normalized to *Renilla* luciferase activity for each well.

### Cell proliferation and colony formation assays

Cell viability was measured using the Cell Counting Kit-8 assay (CCK-8, Dojindo Laboratories, Kumamoto, Japan). HepG2 cells were seeded in 96-well plates at a density of 5 × 10^3^/well and cultured in hAEC-CM. Cell proliferation was detected daily according to the instructions for a total of 7 days. Liver cancer cell lines were seeded in 96-well plates at a density of 5 × 10^3^ cells/well (*n* = 3 for each group). After transfection, the cells were cultured for 1, 2, 3 or 4 days. Subsequently, 10 μl of CCK-8 solution was added to each well and incubated for 4 h at 37 °C. Then, the absorbance value (OD) was measured at 450 nm to calculate the number of viable cells. For the colony formation assay, liver cancer cell lines were plated into 6-well culture plates at a density of 500 cells/well (*n* = 3 for each group). The colonies were washed twice with PBS, fixed with methanol for 30 min and stained with 0.5% crystal violet solution after incubation for 14 days at 37 °C. The colonies composed of more than 50 cells in a well were counted under a microscope (Olympus, Japan). Triplicate experiments were performed for each assay.

### Cell cycle analysis

For cell cycle analysis, a total of 6 × 10^5^ liver cancer cells were harvested and washed twice with cold PBS at 48 h after transfection with miRNA. Then, the cells were fixed with 70% ice-cold ethanol at 4 °C overnight. After incubation with 0.5 ml of propidium iodide/RNase staining buffer (BD Biosciences, USA) for 15 min at room temperature before analysis, BD Accuri C6 flow cytometry (BD Biosciences, USA) was used to analyse the DNA content of labelled cells. The proportions of cells in the G1, S and G2-M phases were analysed using ModFit software.

### Wound healing and invasion assays

The effect of miR-297 on the migration and invasion abilities of HepG2 cells was examined using wound healing and Transwell chamber assays. For the wound healing assay, HepG2 cells were seeded in a six-well plate at a density of 8 × 10^5^ cells/well and cultured in hAEC-CM until reaching 80% confluence. After treatment with 10 μg/mL mitomycin C (Sigma, USA) for 2 h, HepG2 cells were scratched and washed twice with DPBS. The scratches were photographed at 24 h and 48 h. Liver cancer cell lines were seeded in 6-well plates at 1 × 10^6^ cells/well after transfection. Wounds were made by scratching the cell layer using a sterile 200 μl plastic pipette tip. The cells were then washed and cultured with serum-free medium for 72 h at 37 °C with 5% CO_2_. Images were taken by an inverted microscope (Olympus, Tokyo, Japan). For the invasion assay, transwell inserts (BD Falcon, USA) with a diameter of 8 μm were coated with 50 μl of extracellular matrix gel (ECM, Sigma, USA), which was diluted 1:8 with H-DMEM, and incubated at 37 °C for 1 h. After transfection, liver cancer cells (3 × 10^4^ cells/insert) in 200 μl of serum-free H-DMEM were seeded into the upper chambers of the transwells. The chambers were then placed into 24-well plates containing 600 μl of H-DMEM with 20% FBS and incubated for 48 h. The noninvaded cells on the upper surface of the insert were wiped off using a cotton swab, while the invaded cells on the lower surface were fixed in methanol, stained with haematoxylin solution, washed with PBS and counted using an inverted microscope (Olympus, Japan). Five field views were randomly counted and averaged.

### Cell apoptosis assay

Cell apoptosis of liver cancer cell lines was detected using an Annexin V/Dead Cell Apoptosis Kit (Invitrogen, USA). A total of 6 × 10^5^ liver cancer cells were harvested and washed twice with cold PBS at 48 h after transfection. The supernatant was discarded, and the cell pellet was resuspended in 100 μl of 1× binding buffer at a density of 1 × 10^6^ cells/ml after centrifugation. Then, the cells were incubated with 5 μl of FITC-conjugated Annexin V and 5 μl of PI staining solution (100 μg/ml) at room temperature for 15 min in the dark. Finally, 400 μl of 1× binding buffer was added to each sample and analysed by BD Accuri C6 flow cytometry (BD Biosciences, USA).

### In vivo tumorigenesis in nude mice

BALB/c male nude mice at the age of 4 to 6 weeks (Hunan SJA Laboratory Animal CO, China) were used in all studies. The mice were randomly divided into 2 groups (*n* = 6 mice/group) and subcutaneously inoculated in the left hind flank with 1 × 10^6^ logarithmically growing HepG2 cells with or without stable miR-297 overexpression. The expression of miR-297 in HepG2 cells was significantly increased after the transfection of recombinant lentivirus expressing a miR-297 precursor compared with the control lentivirus. All animal experiments were conducted in accordance with the principles and procedures of the Department of Laboratory Animals of Central South University. The procedures for the care and use of animals were approved by the Centre for Medical Ethics, Central South University, and all applicable institutional and governmental regulations concerning the ethical use of animals were followed. The tumour volume was monitored each week and calculated as follows: tumour volume = width^2^ × length. After 6 weeks, all mice were sacrificed, and the tumour tissues were excised and weighed.

### In situ hybridization of miR-297

A tissue microarray containing 70 human liver cancer samples and 20 matched noncancerous tissues adjacent to cancer was purchased from Shanghai Outdo Biotech (Shanghai, China). The tumour samples of tissue microarray were collected from patients with HCC. And the clinicopathologic data of the HCC patients were detailed in Supplementary Table 3. The use of tissues obtained the approval from the ethics committee of Taizhou Hospital of Zhejiang Province. In situ hybridization was performed using a hsa-miR297 probe (3’-DIG labelled) from Boster (Wuhan, China) according to the manufacturer’s protocol using the DAB staining method. The degree of staining was scored according to the staining intensity and positive cell ratio. Scores representing the proportion of positively stained tumour cells were graded as: 0 (<10%); 1 (11–25%); 2 (26–50%); 3 (51–75%) and 4 (>75%). The intensity of staining was graded as: 0 (-); 1 (weak, light yellow); 2 (moderate, yellow brown) and 3 (strong, dark brown). The staining score of miR-297 in the samples was calculated as the product of staining intensity × proportion of positive cell ratio. And the scores were graded as: negative staining (0); low staining (1–4); moderate staining (5–8) and high staining (9–12).

The expression of p53 was detected by immunohistochemistry according to the pathological data provided by Shanghai Outdo Biotech (Shanghai, China). The degree of staining was classified according to the staining intensity and positive cell ratio. Scores representing the proportion of positively stained tumour cells were graded as: 0 (<10%); 1 (11–25%); 2 (26–50%); 3 (51–75%) and 4 (>75%). The intensity of staining was graded as: 0 (-); 1 (weak, light yellow); 2 (moderate, yellow brown) and 3 (strong, dark brown). The staining score of the sample was calculated as the product of staining intensity × proportion of positive cell ratio. According to the expression level of p53, the 70 liver cancer patient samples were divided into p53 positive (1–12) and p53 negative (0) groups.

### Immunohistochemical staining

Immunohistochemistry assays were performed on paraffin-embedded tumour tissues from nude mice. The assays were carried out using a streptavidin-peroxidase kit followed by a DAB substrate kit (Zsbio, Beijing, China) according to the manufacturer’s instructions. Anti-Ki67 antibodies (Cat. No. Ab16667, 1:100, Abcam) were used, and stained tissue sections were visualized under an inverted microscope (Olympus, Japan).

### MiRNA array

A miRNA microarray was used to analyse miRNA expression profile changes in HepG2 cells after intervention. HepG2 cells were seeded with hAEC-CM for 72 h in a six-well plate at a density of 8 × 10^5^ cells/well. The supernatant was discarded, and the cells were washed with DPBS three times. Then, we added 1 ml of TRIzol (Life, USA) per 1 × 10^6^ cells and harvested the cell homogenate in a 1.5 ml EP tube. The EP tubes were labelled and stored at −80 °C. Then, the tubes were kept on dry ice and transported to Beijing for further miRNA array analysis (Beijing CapitalBio Technology Co., Ltd).

### Statistical analysis

SPSS 13.0 software (SPSS, Inc. Chicago, IL, USA) and GraphPad Prism software (version 5.0) were used for statistical analyses. Data are expressed as the mean ± standard derivation (SD) from at least three independent experiments. Comparisons between two groups were performed using Student’s *t*-test and ANOVA for multiple groups (>2). All statistical tests were two-sided, and single, double and triple asterisks indicate statistical significance: *P < 0.05, **P < 0.01 and ***P < 0.001.

## Results

### The expression of miR-297 increased in HepG2 cells after treatment with hAEC-CM

**Fig. 1:**
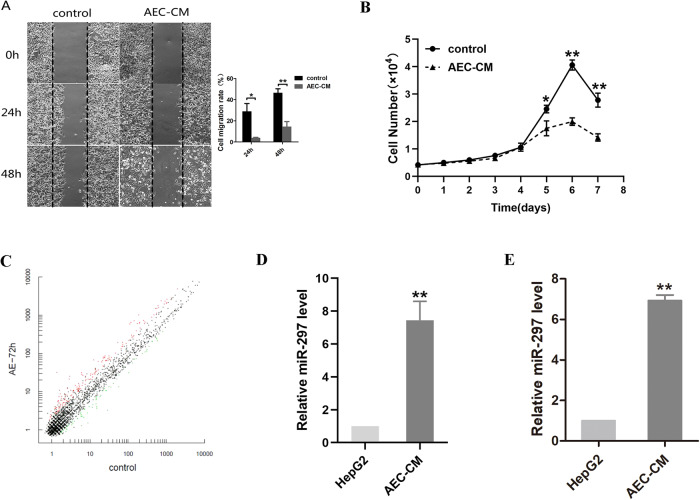
The expression of miR-297 increased in HepG2 after the treatment of hAEC-CM. HepG2 were treated with hAEC-CM for 72 h. **A** Wound healing assays showed the effects on migration of HepG2. **B** Effect of hAEC-CM on cell proliferation was analyzed using CCK-8 assay. **C** The scatter plot of miRNA arrays for hepG2 which was treated for 72 h compared with the control group. **D** The expression of miR-297 in hepG2 was measured by miRNA array. **E** The expression of miR-297 in hepG2 was measured and verified by RT-qPCR. *P < 0.05, **P < 0.01 and ***P < 0.001.

Proliferation and migration abilities were evaluated by CCK-8 and wound-healing assays. Treatment with hAEC-CM significantly inhibited the growth and migration of HepG2 cells (Fig. 1A, B). To determine the mechanism of growth inhibition, we performed miRNA array and RT-qPCR to verify the differential expression of miRNA (Fig. 1C). The expression of miR-297 in hepG2 was measured and verified respectively by miRNA array (Fig. 1D) and RT-qPCR (Fig. 1E). The results of miRNA array showed that the expression of miR-297 increased significantly compared with control group (Fig. 1D). We verified the differential expression of miR-297 in HepG2 after the treatment of hAEC-CM for 72 h by RT-qPCR. The results of RT-qPCR showed that the expression of miR-297 increased significantly in HepG2 cells after the treatment with AEC-CM for 72 h (Fig. 1E).

### MiR-297 inhibits the proliferation, migration and invasion of HCC cell lines in vitro

**Fig. 2:**
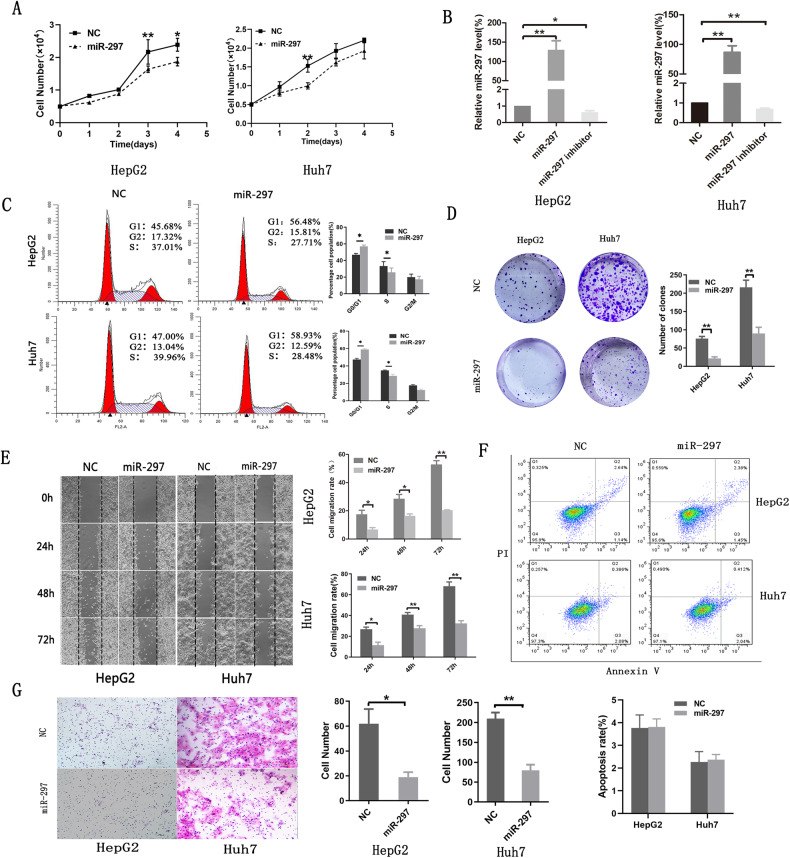
MiR-297 inhibits the proliferation and metastasis of liver cancer cell lines in vitro. The liver cancer cell lines were transfected with 50 nM miR-297 mimic, inhibitor or the negative control miR-NC. **A** Effect of miR-297 on cell proliferation was analyzed using CCK-8 assay. **B** The expression of miR-297 was measured by RT-qPCR. **C** Flow cytometry was performed to determine the effects on cell cycle. **D** Clone formation assays in liver cancer cell lines after transfection. **F** Annexin V/PI staining assays was performed to analyse the apoptosis of liver cancer cell lines. **E**, **G** Wound healing (**E**) and transwell (**G**) assays of cells after transfection showing the effects on migration and invasion of liver cancer cell lines. Scale bar: HepG2: 100 μm; Huh7:200 μm. *P < 0.05, **P < 0.01 and ***P < 0.001.

To determine the effect of miR-297 on the growth of HCC cells in vitro, miR-297 mimics, inhibitors and negative control were introduced into liver cancer cells, including HepG2 and Huh7 cells. The expression of miR-297 in liver cancer cell lines was significantly increased after the transfection of miR-297 mimics compared with the negative control groups by RT-qPCR and decreased significantly in inhibitor-treated liver cancer cells (Fig. 2B).

Proliferation ability was evaluated by CCK-8, colony formation, cell cycle and cell apoptosis assays. The overexpression of miR-297 significantly suppressed cell growth in HepG2 and Huh7 cells (Fig. 2A). The proportion of miR-297-transfected cells at the G0/G1 stage was significantly higher than that of the control group (Fig. 2C), indicating that miR-297 arrested the cell cycle at the G0/G2 stage by suppressing the G2 to S cell cycle transition. Moreover, the number of colonies formed in the transfection group was significantly reduced compared with that in the control group (Fig. 2D). Our results showed that there was no significant difference in cell apoptosis between transfected (miR-297 mimic) and negative control (miR-NC) group (Fig. 2F), as assessed by the proportion of cells that are annexin V positive and propidium iodide negative. The migration and invasion abilities of HepG2 and Huh7 cells were reduced after transfection (Fig. 2E, G). Furthermore, we also evaluated the effects of miR-297 inhibitor on the growth and metastasis of liver cancer cells in vitro. The results showed that miR-297 inhibitor promoted the growth, migration and invasion of liver cancer cells. The results were listed in Supplementary Fig. 1. These results confirm that miR-297 inhibits the proliferation, migration and invasion of liver cancer cells in vitro.

### Overexpression of miR-297 suppressed the in vivo tumour growth of HepG2 cells

**Fig. 3:**
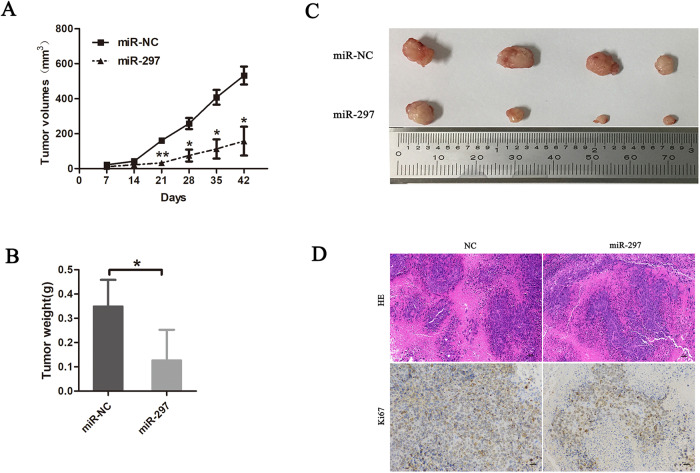
Male BALB/C mice (4–6 weeks) were subcutaneously injected with 1 × 10^6^ logarithmically growing HepG2 cells transfected with a control lentivirus of a recombinant lentivirus expressing a miR-297 precursor (*n* = 6 mice/group). **A** Tumour volumes was measured for each mouse every seven days, and tumour growth curves was plotted. **B** The effect of miR-297 in vivo was evaluated in xenograft mouse models bearing tumours originating from HepG2. **C** Tumour weight was measured at 6 weeks after subcutaneous injection. **D** Representative H&E as well as Ki67 IHC staining of primary Tumour tissues were shown. Scale bar: 50 μm. *P < 0.05 and **P < 0.01.

As miR-297 inhibits cell growth in HepG2 and Huh7 cells in vitro, we conducted an in vivo tumour formation experiment. We evaluated the in vivo antitumour efficacy of miR-297 by subcutaneously injecting HepG2-miR-297 cells and control cells into nude mice. The tumour volumes were measured each week, and the mice were sacrificed 6 weeks after tumour implantation. From day 21, the tumour volumes were significantly lower in miR-297 group than the control group (Fig. 3A). At the end of the experimental period, the mean weight of tumours in miR-297 group was significantly lower than in control group (Fig. 3B, C). The expression of Ki67 was weaker than that in the tumours of the control group (Fig. 3D). These results suggested that miR-397 significantly inhibits tumorigenesis in vivo.

### Expression of miR-297 in human liver cancer patient tissues

**Fig. 4:**
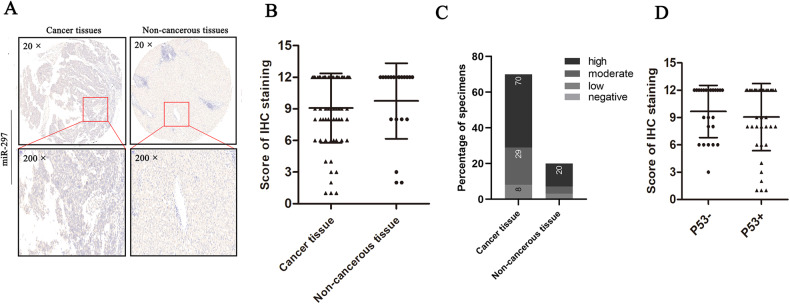
The expression of miR-297 in liver cancer patient tissues. **A** Representative IHC staining of miR-297 in liver cancer patient tissues and adjacent non-cancerous tissues. **B** MiR-297 levels have a tendency to decrease in the cancer tissues compared with the noncancerous tissues, but there are no statistically significant differences. **C** The proportion of the four groups: negative staining; low staining; moderate staining and high staining between the cancer and non-cancerous tissues showed no significant difference. **D** The expression of miR-297 in p53 negative(p53-) and positive(p53+) group.

To investigate the expression pattern of miR-297 in liver cancer patient tissues, we performed a tissue microarray containing 70 human liver cancer patient samples and 20 matched noncancerous tissues adjacent to cancer. The results indicated that miR-297 levels have a tendency to decrease in the cancer tissues compared with the noncancerous tissues, but there are no statistically significant differences. (*P* > 0.05; Fig. 4A, B). This is consistent with what has been found in previous studies which showed that the expression of miR-297 reduced significantly in tissues from the HCC patients compared with normal liver tissues [16]. And we compared the proportion of the four groups of miR-297 expression: negative staining (0); low staining (1–4); moderate staining (5–8) and high staining (9–12) in the cancer and non-cancerous tissues. The results showed no significant difference in the proportion of the four groups between the cancer and non-cancerous tissues (Fig. 4C). According to the expression level of p53, the 70 liver cancer patient samples were divided into p53 positive (1–12) and p53 negative (0) groups. We compared the two groups and found that the levels of miR-297 were lower in the positive group (Fig. 4D).

### MiR-297 targets and down-regulates PTBP3 to inhibit the PI3K/AKT signaling pathway

**Fig. 5:**
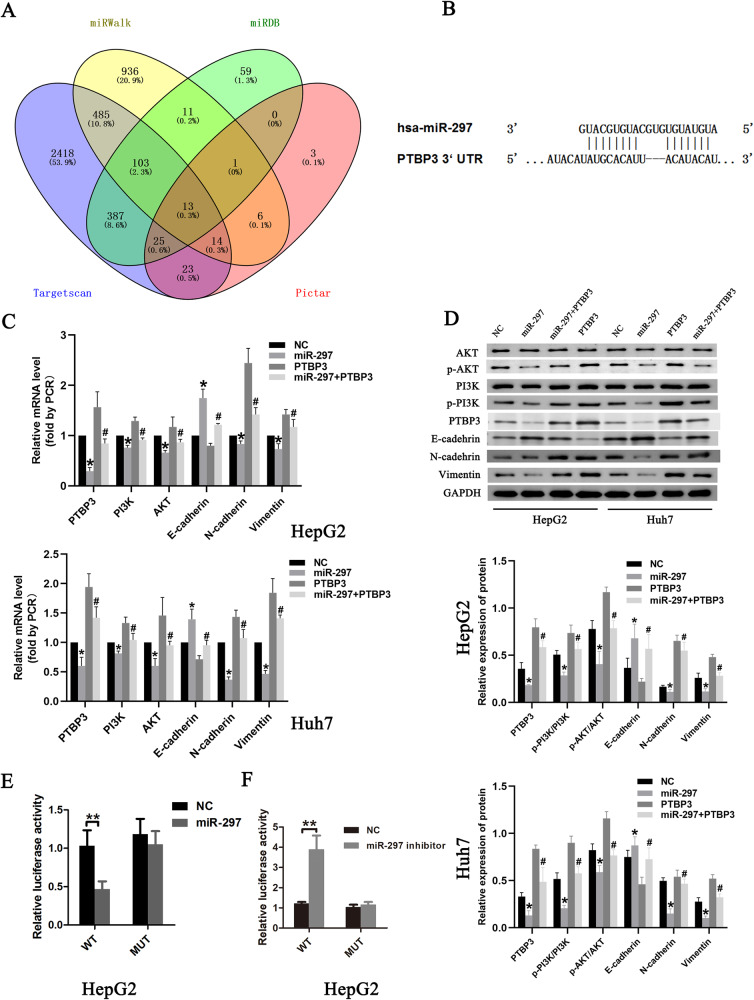
MiR-297 targets and down-regulates PTBP3 to inhibit the PI3K/AKT signaling pathway. **A** The candidate target genes of miR-297 was predicted by four different prediction algorithms: Targetscan, PicTar, miRWalk and miRDB. **B** The predicted binding sites of miR-297 in the 3’ UTR of PTBP3 and corresponding mutant sites of PTBP3. **C** RT-qPCR detection of the expression of PTBP3, PI3K/AKT signaling pathway-related factors and EMT related markers in HepG2 and Huh7 cells transfected with hsa-miR-297 mimic or PTBP3 plasmid. **D** Western blot analysis of protein expression of PTBP3, PI3K/AKT signaling pathway-related factors and EMT related markers in HepG2 cells and Huh7 cells transfected with hsa-miR-297 mimic or PTBP3 plasmid. *p < 0.05 vs. the NC treatment. #*p* < 0.05 vs. HCC cells transfected with hsa-miR-297 mimic. Cell experiments were repeated for three times. **E**, **F** The Luciferase reporter assays was conducted at 48 h after co-transfection of HepG2 cells with the PTBP3 WT/PTBP3 MUT plasmid and hsa-miR-297 mimics or inhibitor. *P < 0.05, **P < 0.01 and ***P < 0.001.

Next, we searched four online miRNA target bioinformatics prediction databases (TargetScan, PicTar, miRWalk and miRanda) to identify the candidate target genes of miR-297. Initially, 13 potential genes of miR-297 were predicted in all four databases (Fig. 5A). PTBP3 were predicted to be a direct target of miR-297 by all of the four databases and the expression of PTBP3 showed higher expression in tumor tissues compared with their matched adjacent normal tissues in liver hepatocellular carcinoma according to TCGA database (Supplementary Fig. 6). A complementary miR-297 sequence was identified in the 3’-UTR of the PTBP3 mRNA (Fig. 5B). Overexpression of miR-297 downregulated PTBP3 mRNA and protein levels in HepG2 cells (Fig. 5C, D). To verify PTBP3 was an authentic downstream target of miR-297, we conducted a dual-luciferase reporter assay. Dual reporter assays demonstrated that the transfection of the miR-297 mimic significantly decreased PTBP3 luciferase reporter activity (Fig. 5E), while the miR-297 inhibitor had the opposite effect (Fig. 5F). However, the miR-297 mimic did not suppress the luciferase activity of PTBP3 when cotransfected with the mutated PTBP3 reporter. The results highlighted the bind of PTBP3-3’-UTR by miR-297.

PTBP3 was highly expressed in most tumours and the overexpression of PTBP3 generally predicts poor overall survival and disease-free survival in patients of with adrenocortical carcinoma, lung squamous cell carcinoma, and pancreatic adenocarcinoma [17]. Other studies have revealed that PTBP3 knockdown in gastric cancer cells inhibited the phosphorylation of Akt, which has also been demonstrated to be involved in HCC growth and metastasis [18]. The previous reports suggest that the role of PTBP3 in HCC is most likely realized associated with the phosphorylation of Akt. The previous reports suggest that the role of PTBP3 in HCC is most likely realized associated with the phosphorylation of Akt. For validation, the expression of AKT, phosphorylated AKT(p-AKT), PI3K, phosphorylated(p-PI3K) were determined by western blotting. We found that miR-297 mimic treatment led to increased miR-297 but reduced PTBP3, p-PI3K, AKT and p-AKT expression as well as p-AKT/AKT ratio; while PTBP3 plasmid transfection caused increased PTBP3, p-PI3K, AKT and p-AKT expression as well as p-AKT/AKT ratio. Relative to miR-297 mimic treatment, miR-297 mimic+PTBP3 plasmid treatment exerted no effect on miR-297 expression but increased PTBP3, p-PI3K, and p-AKT expression as well as p-AKT/AKT ratio (Fig. 5D). These results suggested that miR-297 could specifically bind to the PTBP3-3’-UTR to downregulate PTBP3 expression, thereby suppressing PI3K/AKT signaling pathway.

Existing studies show that PTBP3 overexpression induced epithelial-mesenchymal transition (EMT) and promotes the invasive growth and metastasis of breast tumor cells and lung adenocarcinoma cells [19, 20]. To explore the mechanisms by which miR-297 suppresses HCC cell migration and invasion, western-blot analysis of EMT-related genes were performed. We found that miR-297 overexpression downregulated PTBP3, mesenchymal markers (N-cadherin and Vimentin) but enhanced epithelial markers(E-cadherin), while PTBP3 plasmid transfection caused increased PTBP3, N-cadherin and Vimentin expression but reduced E-cadherin expression (Fig. 5D). MiR-297 mimic+PTBP3 plasmid treatment restored the levels of EMT-related levels. Taken together, PTBP3 overexpression induced EMT in HepG2 and Huh7 cells. Overexpression of miR‑297 in HCC cells could inhibit EMT by downregulating PTBP3 expression.

### Decreased PTBP3 expression by miR-297 inhibits the PI3K/AKT signaling pathway and suppresses the proliferation, migration, and invasion of HCC cell lines in vitro

**Fig. 6:**
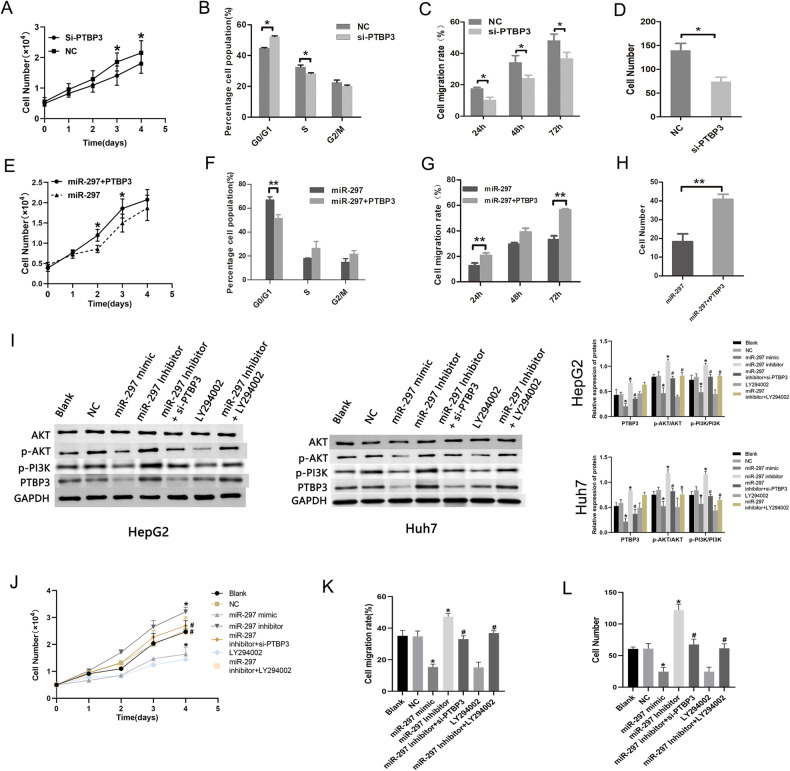
Decreased PTBP3 expression blocks the PI3K/AKT signaling pathway and depresses proliferation, migration, and invasion in HCC cells. HCC cell lines were treated with miR-297 mimic, miR-297 inhibitor, NC, LY294002, miR-297 inhibitor + si-PTBP3, or miR-297 inhibitor + LY294002. CCK-8 assays (**A**), cell cycle distribution (**B**), wound healing (**C**) and transwell assays (**D**) of HepG2 cells transfected with si-PTBP3 or si-NC were performed. **E**–**H** Overexpression of PTBP3 reversed the suppression of miR-297 overexpression on the proliferation (**E**), migration (**F**) and invasion (**G**) and the cell cycle arrest effect (**H**) of HepG2 cells. **I** Western blot analysis of protein expression of PTBP3 and PI3K/AKT signaling pathway-related factors in HCC cell lines. CCK-8 assays (**J**), wound healing (**K**) and transwell assays (**L**) of HepG2 cells transfected with treated with miR-297 mimic, miR-297 inhibitor, NC, LY294002, miR-297 inhibitor + si-PTBP3, or miR-297 inhibitor + LY294002 were performed. *p < 0.05 vs. HCC cells treated with NC. #*p* < 0.05 vs. HCC cells treated with miR-297 inhibitor. The cell experiments were repeated 3 times. *P < 0.05, **P < 0.01 and ***P < 0.001.

After confirming PTBP3 is a direct target of miR-297 in HepG2 cells, we further explored the role of miR-297-mediated PTBP3 in HCC biological functions in relation to PI3K/AKT signaling pathway. We first transfected si-PTBP3 and a negative control(si-NC) into HepG2 cells to determine whether decreased PTBP3 could inhibit the proliferation, migration and invasion of tumour cells. The obtained results showed that the transfection of si-PTBP3 inhibited the proliferation (Fig. 6A), migration (Fig. 6C) and invasion (Fig. 6D) of HepG2 cells. And the cell cycle was arrested at the G0/G1 stage (Fig. 6B). To further confirm the role of PTBP3 in the suppression function of miR-297 on HepG2 cells, we cotranfected miR-297 mimic and PTBP3 plasmid to HepG2 cells to determine whether the overexpression of PTBP3 could reverse the inhibitory effects of miR-297 overexpression on the progression of HepG2 cells. The results demonstrated that the overexpression of PTBP3 reversed the suppression of miR-297 overexpression on the proliferation (Fig. 6E), migration (Fig. 6G) and invasion (Fig. 6H) and the cell cycle arrest effect (Fig. 6F) of HepG2 cells. The results demonstrated that knockdown of PTBP3 inhibited the growth and metastasis of HepG2 cells, and overexpression of PTBP3 rescued the suppression effects of miR-297 overexpression on the progression of the cells.

To explore the role of miR-297-mediated PTBP3 in HCC cell functions in relation to PI3K/AKT signaling pathway, the expression of PTBP3, p-PI3K, AKT and p-AKT were evaluated by western blot in HepG2 and Huh7 cells after the transfection of miR-297 mimics, miR-297 inhibitor. As reflected by the results of western blot analysis, the p-PI3K, p-AKT and PTBP3 expression as well as p-AKT/AKT ratio was decreased in HepG2 and Huh7 cells after treatment of miR-297 mimic, whereas the opposite trends were observed in miR-297 inhibitor-treated HCC cells. Moreover, relative to miR-297 inhibitor treatment, further LY294002 treatment did not affect PTBP3 expression but caused a decline in p-PI3K and p-AKT expression as well as p-AKT/AKT ratio in HepG2 and Huh7 cells, while miR-297 inhibitor + si-CREB5 treatment caused reductions in p-PI3K, p-AKT and PTBP3 expression (Fig. 6I). CCK-8 assays noted that at 3 and 4 days, miR-297 inhibitor induced cell growth while LY294002 or miR-297 mimic treatment led to opposite trend; relative to inhibitor treatment, miR-297 inhibitor + si-PTBP3 or miR-297 inhibitor + LY294002 treatment suppressed cell proliferation of HCC cell lines (Fig. 6J). Additionally, miR-297 inhibitor induced invasiveness, and migration of HCC cells while LY294002 or miR-297 mimic transfection led to opposite trend; relative to miR-297 inhibitor treatment, miR-297 inhibitor + si-PTBP3 or miR-297 inhibitor + LY294002 treatment suppressed invasion, and migration of HCC cells (Fig. 6K, L). Taken together, these results support that miR-297 overexpression depressed HCC cell proliferation, migration and invasion through suppression of the PI3K/AKT signaling pathway by decreasing PTBP3.

## Discussion

Despite significant progress in liver cancer diagnosis and therapy, the 5-year survival rate for liver cancer remains poor due to the high rate of recurrence and malignant proliferation. Therefore, poor prognosis and outcomes may be improved by clarification of the mechanism of tumour development in liver cancer and the identification of an effective target for therapy. MiR-297 has been reported to be linked with various types of cancer [13–16]. However, the roles and mechanisms of miR-297 in liver cancer have not been reported. In this study, we demonstrated that miR-297 inhibited the proliferation and G1/S cell cycle transition of HepG2 and Huh7 cells in vitro. It also suppressed the invasion and migration of liver cancer cells. According to the effects of miR-297 on liver cancer in vitro, we conducted an in vivo tumour formation experiment with HepG2 cells and found that miR-297 also suppressed the tumorigenicity of HepG2 cells in vivo. These results suggest that miR-297 functions as a potential tumour suppressor in liver cancer.

In clinical samples, miR-297 levels have a tendency to decrease in the cancer tissues compared with the noncancerous tissues. This is consistent with what has been found in previous studies which showed that the expression of miR-297 reduced significantly in tissues from the HCC patients compared with normal liver tissues [16]. To confirm whether the expression of miR-297 is correlated with the expression of p53, the seventy liver cancer patient samples were divided into p53 positive (1–12) and p53 negative (0) groups according to the expression level of p53. The levels of miR-297 were lower in the positive group, but this result was not statistically significant which is not enough for us to confirm whether miR-297 is negatively correlated with p53 expression. As the wild-type p53 protein detection is susceptible to the influence of half-life while the mutant type detection is relatively stable, we speculated whether methodological limitations influenced the results of the correlation and if the mRNA detection is a better way to investigate the expression.

Polypyrimidine tract binding protein 3 (PTBP3) plays a critical role in post-transcriptional regulation including alternative splicing (AS) regulation, maturation, transport, translation, RNA decay, storage and turnover [17–19, 21, 22]. PTBP3 was highly expressed in most tumours, such as breast invasive carcinoma, colon adenocarcinoma and hepatocellular carcinoma. PTBP3 overexpression generally predicts poor overall survival and disease-free survival in patients with adrenocortical carcinoma, lung squamous cell carcinoma, and pancreatic adenocarcinoma. Although the role of PTBP3 in various human tumours was explored, the molecular function and mechanism of PTBP3 in hepatocellular carcinoma are still lacking. PTBP1 is one of the most thoroughly investigated RBPs and plays a comprehensive role in cancer initiation, progression and cell apoptosis [23–25]. Recent studies have shown that PTBP3 promotes epithelial–mesenchymal transition in breast cancer through the regulation of ZEB1 mRNA stability [20]. Chen et al. demonstrated that PTBP3 may promote proliferation and inhibit the differentiation of MKN45 human gastric cancer cells [2]. In this study, PTBP3 was identified as a direct target gene of miR-297 in liver cancer through dual-luciferase reporter gene experiments and the effect of PTBP3 knockdown on HepG2 cells was investigated by cell function assays. The results demonstrated that decreased PTBP3 inhibited the growth, migration and invasion of HepG2 cells, and the overexpression of PTBP3 rescued the suppression effects of miR-297 overexpression on the progression of HepG2 cells.

Other studies have revealed that PTBP3 knockdown in gastric cancer cells inhibited the phosphorylation of Akt, which has also been demonstrated to be involved in HCC growth and metastasis [18]. The PI3K/AKT signaling pathway is overactive and controls several important cellular processes, including cell growth, survival regulation, and drug resistance, in multiple solid tumors [26]. The PI3K/AKT signaling pathway is the most critical and hyperactivated pathway in the development of HCC [27]. Various studies have reported a strong association between the PI3K/AKT signaling pathway and HCC and the inhibition of the pathway could be a viable HCC treatment. The previous reports suggest that the role of PTBP3 in HCC is most likely realized associated with the phosphorylation of Akt. In subsequent experiments, we also found that miR-297 overexpression repressed cell proliferation, invasion, colony formation and migration in HCC by inhibiting the PI3K/AKT signaling pathway via PTBP3. The knockdown of PTBP3 inhibited the growth and metastasis of HepG2 cells, and overexpression of PTBP3 rescued the suppression effects of miR-297 overexpression on the progression of the cells. EMT is a critical process in the invasion and metastasis of liver cancer. It is involved in the upregulation of mesenchymal markers such as vimentin and snail and the downregulation of epithelial markers such as E-cadherin [28–30]. Recent studies have defined PTBP3 as a regulator of EMT that acts by governing expression of ZEB1, which establish an oncogenic function of PTBP3 in breast tumor cells [31]. In the present study, PTBP3 was shown to support the proper function of the EMT transcription program, acting to down-regulate epithelial markers (E-cadherin), but up-regulating mesenchymal markers (Vimentin and N-cadherin). MiR-297 overexpression induces the reduction of PTBP3 expression and thus inhibits the EMT-like process in HCC cell lines.

The limitation of the study was that the inhibition of metastasis should be clarified in vivo. The 3’ UTR of PTBP3 combined with miR-297 in liver cancer should be mutated to confirm that the inhibitory effect is mediated by PTBP3. Taken together, our studies showed that miR-297 inhibits the proliferation, migration and invasion of liver cancer cells. PTBP3 was identified as a direct target gene of miR-297 in liver cancer and mediated the function of miR-297 in liver cancer cells. miR-297 and PTBP3 might act as potential therapeutic targets for liver cancer.

## Conclusion

In the present study, we demonstrated that the overexpression of miR-297 inhibits the proliferation, migration and invasion of liver cancer cells in vitro and suppresses the tumorigenesis of liver cancer cells in vivo. PTBP3 was identified as a direct target gene of miR-297 in liver cancer cells. Mir-297 could target PTBP3 directly and inactivate the PI3K/AKT signaling pathway to suppress the growth, migration and invasion of HCC cell lines. Therefore, miR-297 might be an effective potential therapeutic target for liver cancer.

## Supplementary information

Supplementary materials

Supplementary Fig.1

Supplementary Table 3

Original Western blotting images

Report summary

## Data Availability

The datasets used and/or analyzed during the current study are available from the corresponding author upon reasonable request.
