# Supplementary material for: MiR-297 inhibits tumour progression of liver cancer by targeting PTBP3
Source: Cell Death Dis. 2023 Aug 26;14(8):564. doi: 10.1038/s41419-023-06097-0 (PMC10460384; doi:10.1038/s41419-023-06097-0)
Supplement: Supplementary file 1 — Supplementary materials [file 41419_2023_6097_MOESM1_ESM.docx]

**Supplementary Table 1：The sequences of siRNA in this study**

| Gene | No. | Target Sequence |
| --- | --- | --- |
| PTBP3 | 1 | GGATGGCCAGAATATCTAT |
|  | 2 | GAGACTTCACTCGCTTAGA |
|  | 3 | AAGCCGGGCTCTAAAAACT |

**Supplementary Table 2: The primers used for RT-PCR in this study**

| Primer names | Sequences |
| --- | --- |
| GAPDH | F:5'-GAAGGTGAAGGTCGGAGTC-3' |
|  | R:5'-GAAGATGGTGATGGGATTTC-3' |
| PTBP3 | F:5'-GGGGTCTCGCCAACTC-3' |
|  | R:5'-CGCATCGTCACGCTGT-3' |
| E-cadherin | F:5'-GACAACAAGCCCGAATT-3' |
|  | R:5'-GGAAACTCTCTCGGTCCA-3' |
| AKT | F:5'-TCTATGGCGCTGAGATTGTG-3' |
|  | R:5'-CTTAATGTGCCCGTCCTTGT-3' |
| PI3K | F:5'-TGTGGCACAGACTTGGTGTT-3' |
|  | R:5'-TTCTTCCCTTGAGATGTCTCCC-3' |
| N-cadherin | F:5'-TGTGGATTTACCTTATCCCCTCA-3' |
|  | R:5'-GTTTGGCTTTGGTCGTTCTGT-3' |
| vimentin | F:5'-GAGAACTTTGCCGTTGAAGC-3' |
|  | R:5'-GCTTCCTGTAGGTGGCAATC-3' |
| U6 | F:5'-CTCGCTTCGGCAGCACATATA-3' |
| miR-297 | F:5'-ATGTATGTGTGCATGTGCATG-3' |

**Supplementary Figure 1**


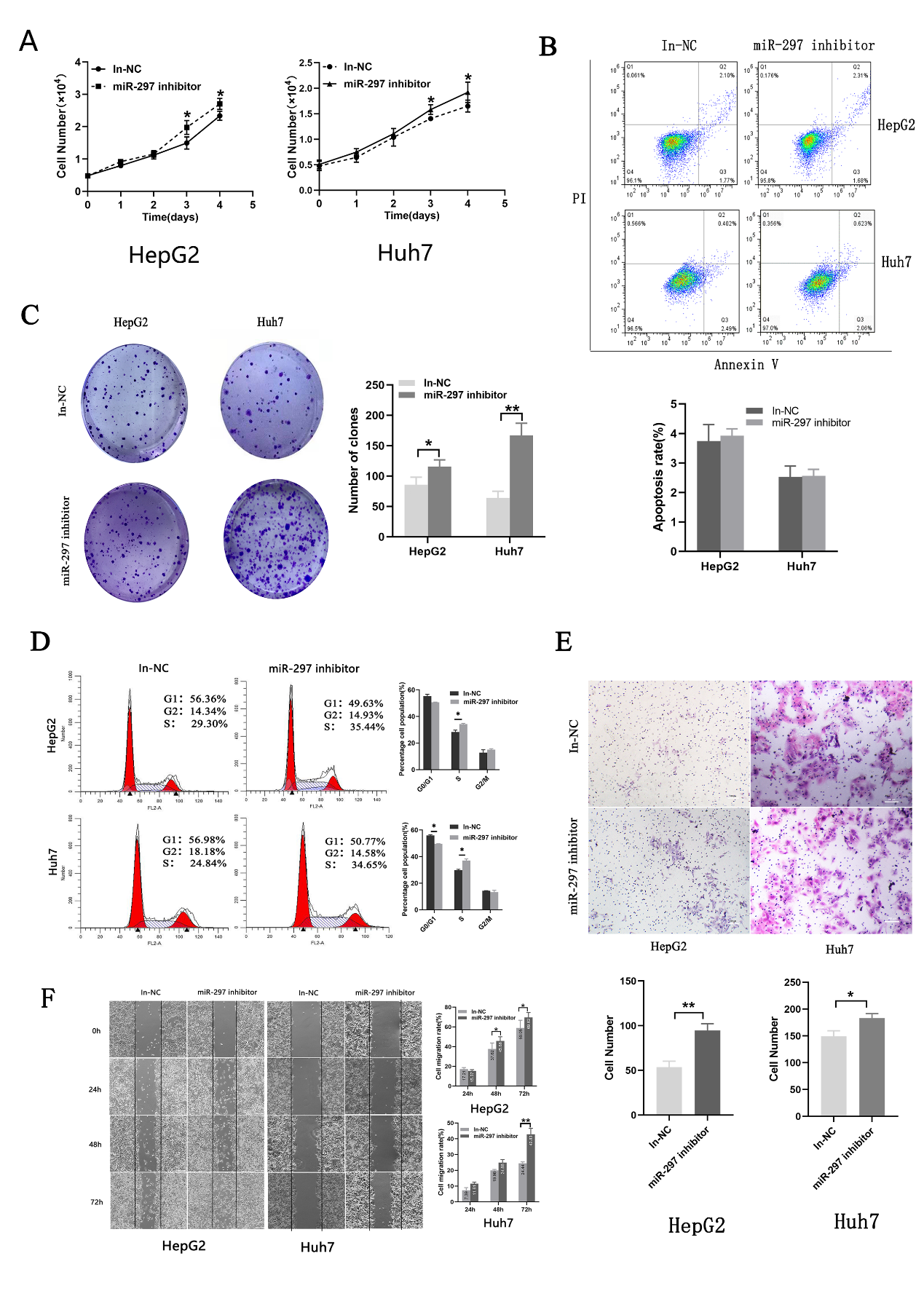


**Supplementary Fig. 1** MiR-297 inhibitor promotes the proliferation and metastasis of liver cancer cell lines in vitro. The liver cancer cell lines were transfected with 100nM miR-297 inhibitor or the negative control In-NC. a. Effect of miR-297 inhibitor on cell proliferation was analyzed using CCK-8 assay. b. Annexin V/PI staining assays was performed to analyze the apoptosis of liver cancer cell lines. c. Clone formation assays in liver cancer cell lines after transfection. d. Flow cytometry was performed to determine the effects on cell cycle. e，f. Wound healing(f) and transwell(e) assays of cells after transfection showing the effects on migration and invasion of liver cancer cell lines. Scale bar: HepG2: 100μm; Huh7:200μm. *P＜0.05, **P＜0.01 and ***P＜0.001.

**Supplementary Figure 2 Original Western blotting images of Figure 5**

**
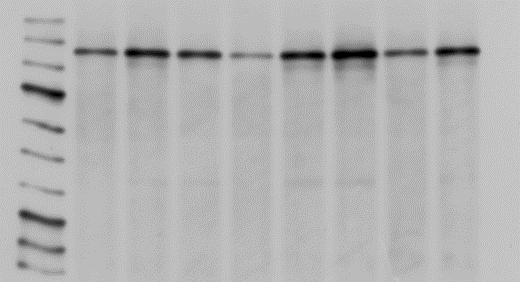

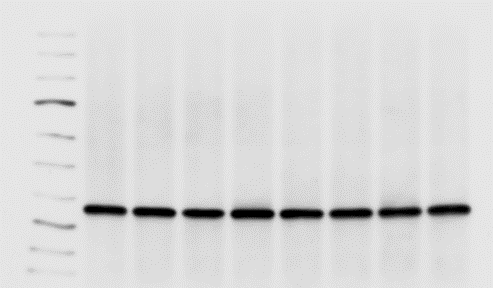
**

**GAPDH 36KD**

**E-cadherin 135KD**


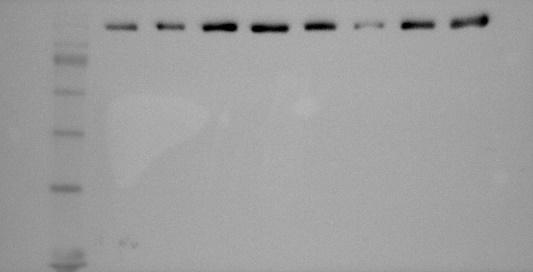

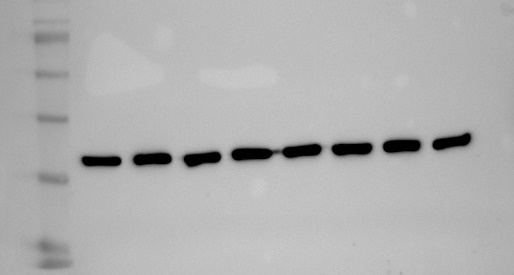


**N-cadehrin 130KD**

**GAPDH 36kD**


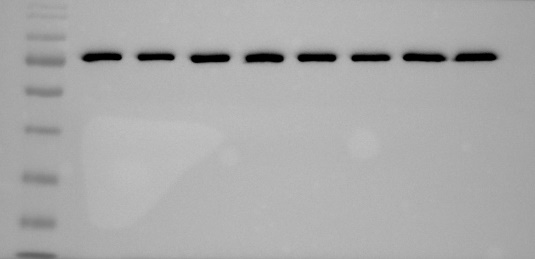

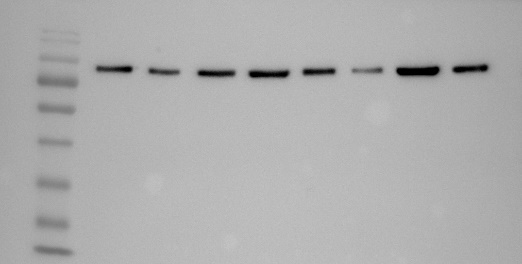


**p-PI3K 84KD**

**PI3K 83KD**


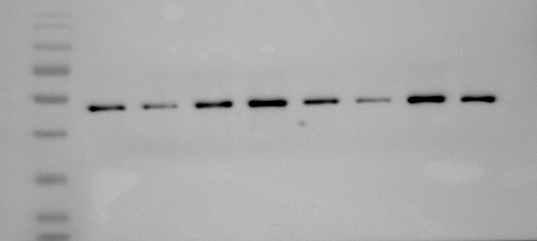
 **
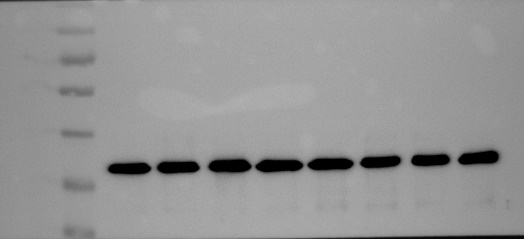
**

**GAPDH 36KD**

**Vimentin 54KD**

**
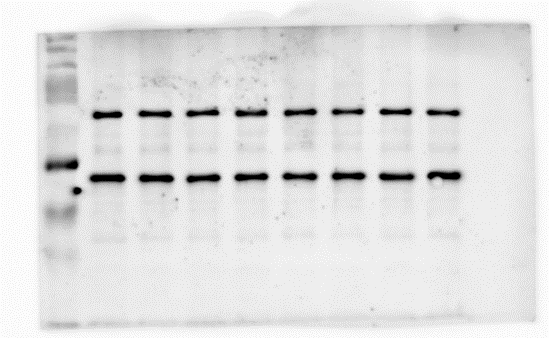

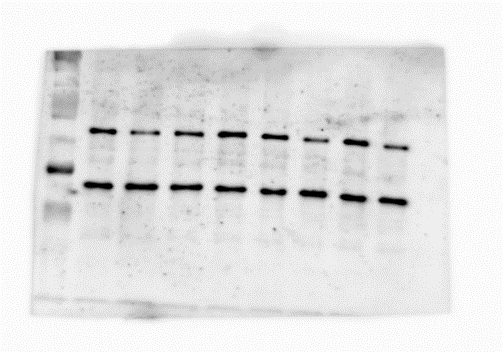
**

**p-AKT 55KD**

**AKT 56KD**

**GAPDH 36KD**

**GAPDH 36KD**

**
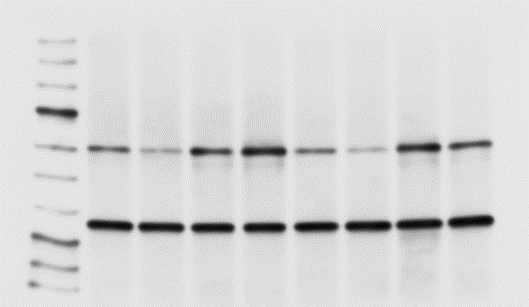
**

**GAPDH 36KD**

**PTBP3 60KD**

**Supplementary Figure 3 Original Western blotting images of Figure 6**

**
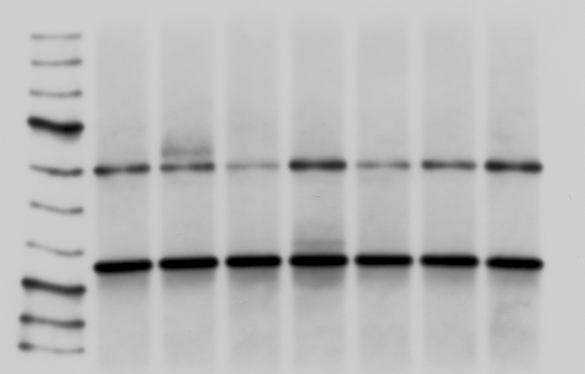

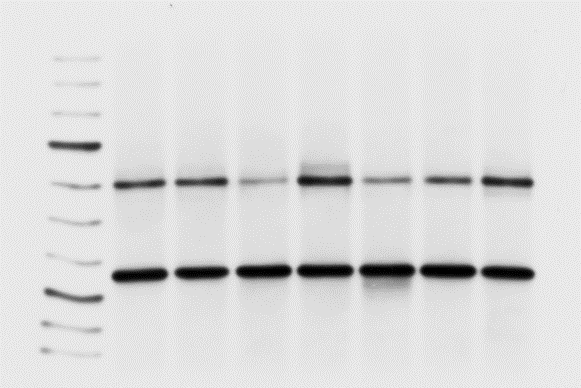
**

**PTBP3 60KD**

**GAPDH 36KD**

**PTBP3 60KD**

**GAPDH 36KD**

**
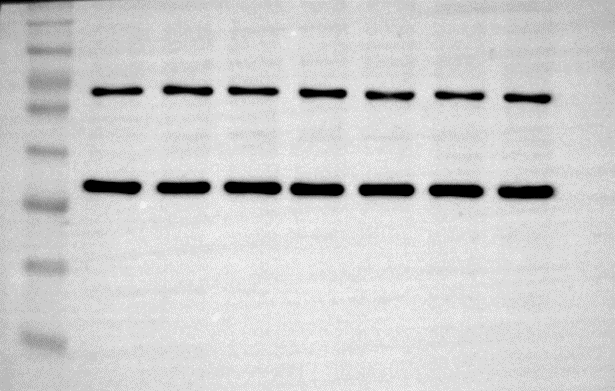

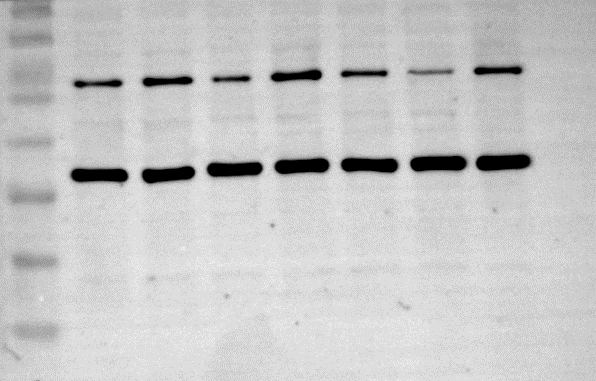
**

**GAPDH 36KD**

**P-AKT 55KD**

**GAPDH 36KD**

**AKT 56KD**

**
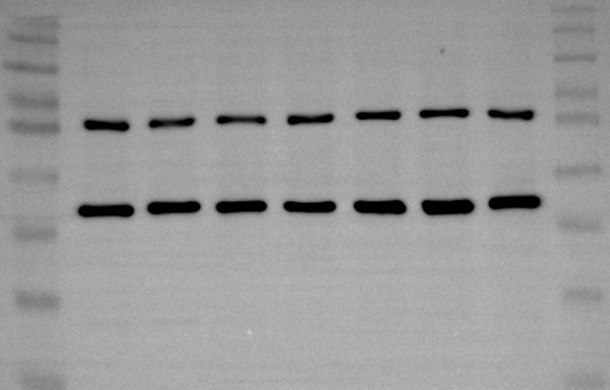

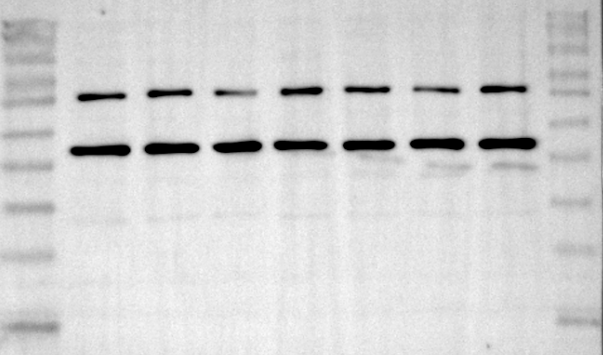
**

**GAPDH 36KD**

**P-AKT 55KD**

**GAPDH 36KD**

**AKT 56KD**

**
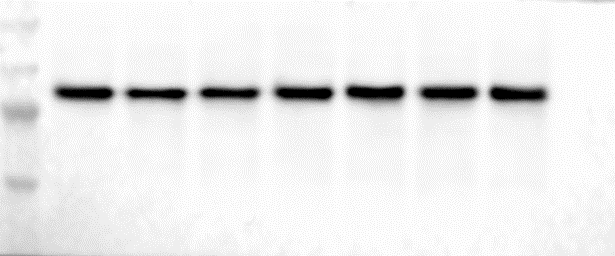

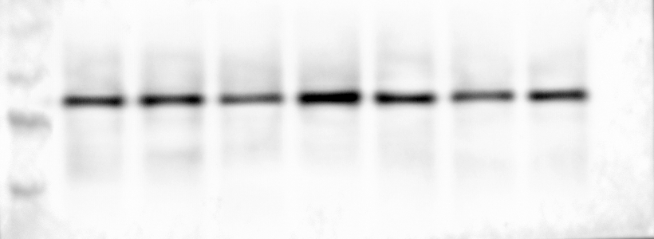
**

**p-PI3K 84KD**

**PI3K 83KD**

**
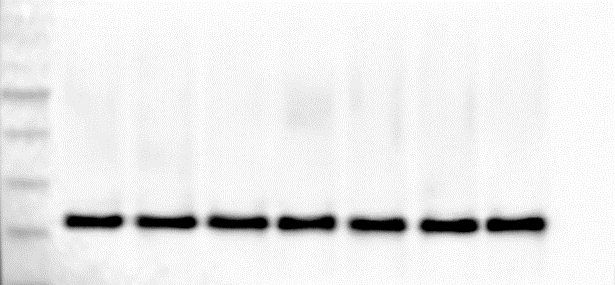

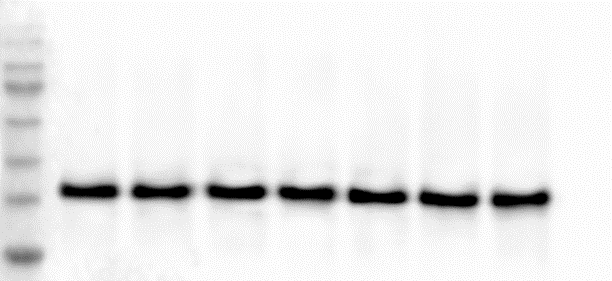
**

**GAPDH 36KD**

**GAPDH 36KD**

**
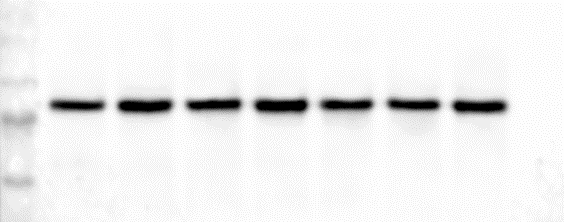

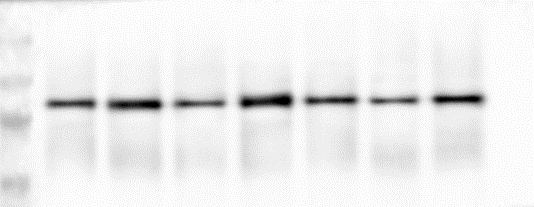
**

**p-PI3K 84KD**

**PI3K 83KD**

**
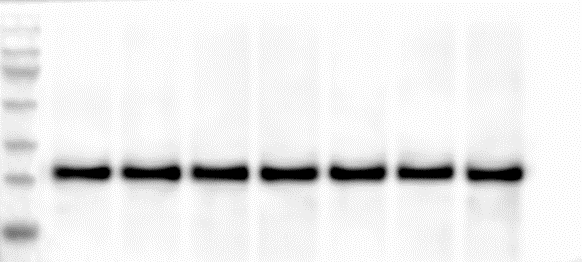

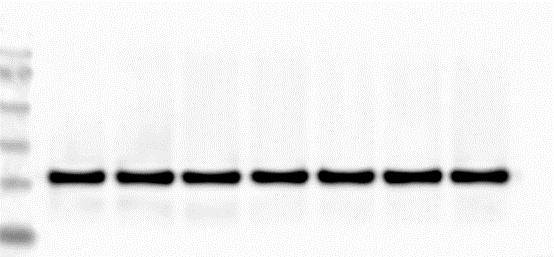
**

**GAPDH 36KD**

**GAPDH 36KD**

**Supplementary Figure 4 Average number of cells per colony**


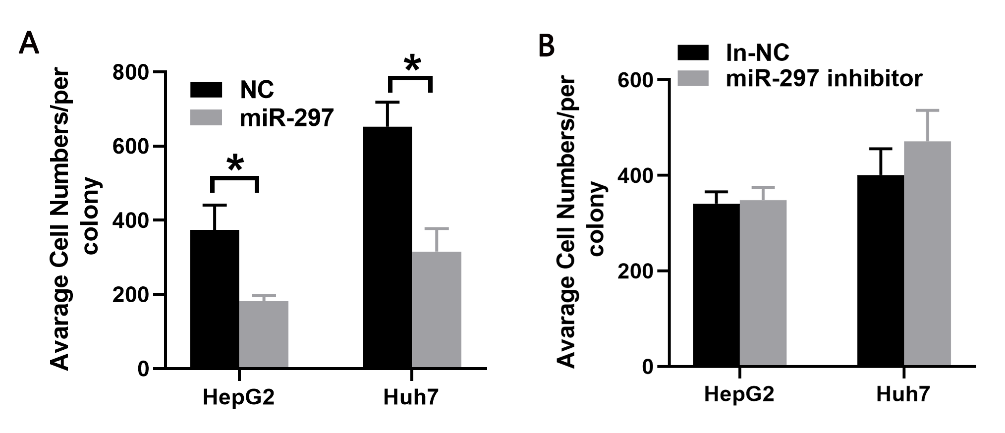


**Supplementary Fig.4** The average number of cells per colony of HepG2 cells and Huh7 cells transfected with miR-297 or miR-297 inhibitor. a. The average number of cells per colony of HepG2 cells and Huh7 cells transfected with miR-297 or NC. b. The average number of cells per colony of HepG2 cells and Huh7 cells transfected with miR-297 inhibitor or in-NC.

**Supplementary Figure 5**


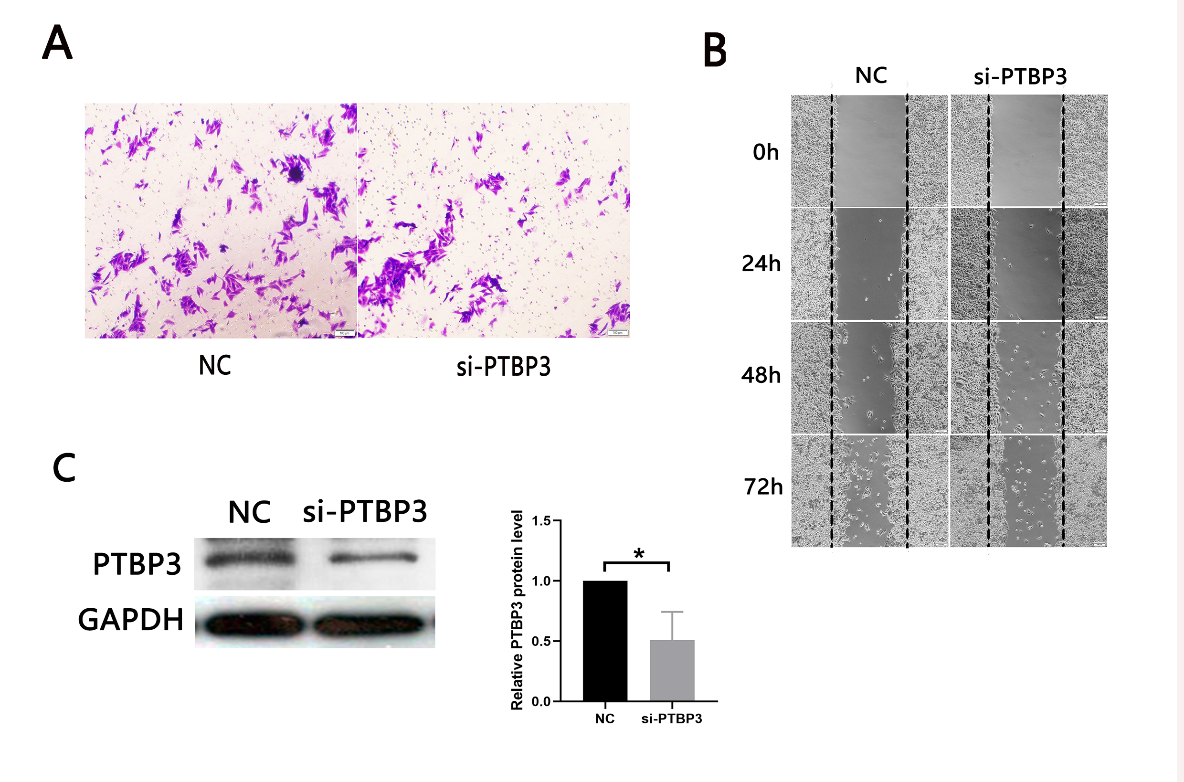


**Supplementary Fig.5** Wound healing and transwell assays of HepG2 cells transfected with si-PTBP3 or si-NC were performed. The transfection of si-PTBP3 decreased the expression of PTBP3 protein in hepG2 cells(c). Overexpression of PTBP3 reversed the suppression of miR-297 overexpression on the invasion (a) and migration (b) of HepG2 cells.

**Supplementary Figure 6**

**
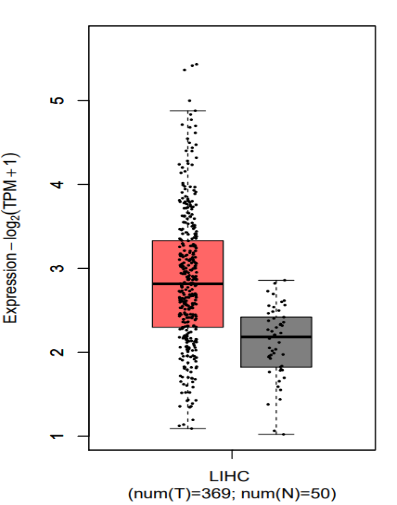
**

**Supplementary Fig.6** The expression of PTBP3 showed higher expression in tumor tissues compared with their matched adjacent normal tissues in liver hepatocellular carcinoma according to TCGA database.
