# Supplementary figures and images for: MiR-297 inhibits tumour progression of liver cancer by targeting PTBP3
Source: Cell Death Dis. 2023 Aug 26;14(8):564. doi: 10.1038/s41419-023-06097-0 (PMC10460384; doi:10.1038/s41419-023-06097-0)

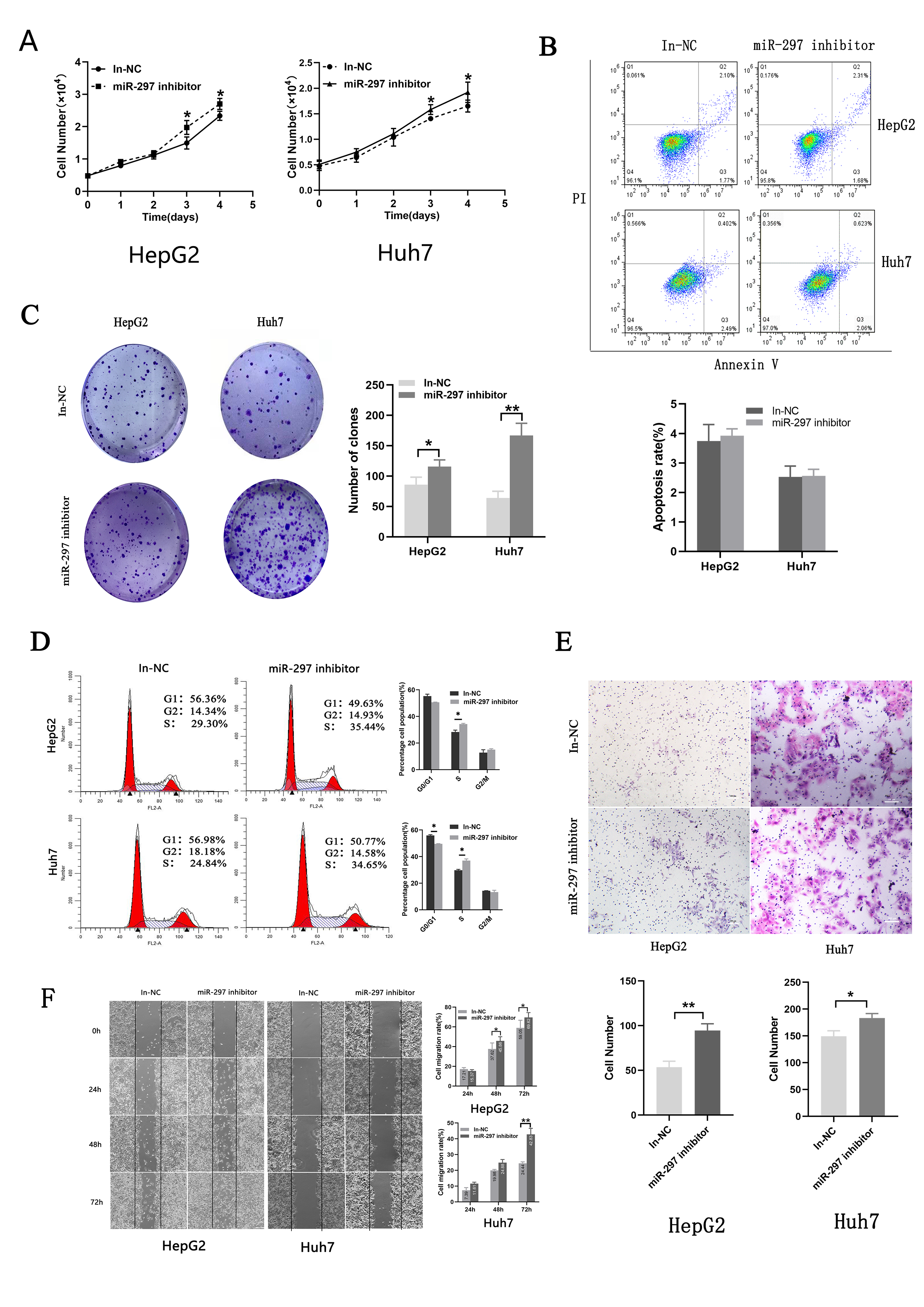

Supplement: Supplementary file 2 — Supplementary Fig.1 [file 41419_2023_6097_MOESM2_ESM.tif]
