# Supplementary material for: MiR-297 inhibits tumour progression of liver cancer by targeting PTBP3
Source: Cell Death Dis. 2023 Aug 26;14(8):564. doi: 10.1038/s41419-023-06097-0 (PMC10460384; doi:10.1038/s41419-023-06097-0)
Supplement: Supplementary file 4 — Original Western blotting images [file 41419_2023_6097_MOESM4_ESM.docx]

**Supplementary Figure 2 Original Western blotting images of Figure 5**

**
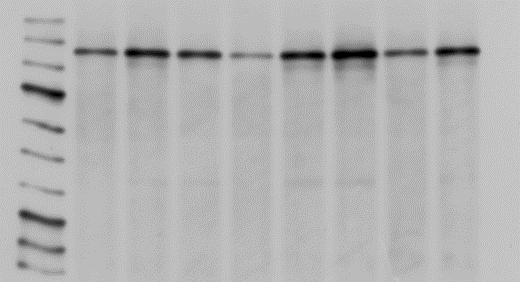

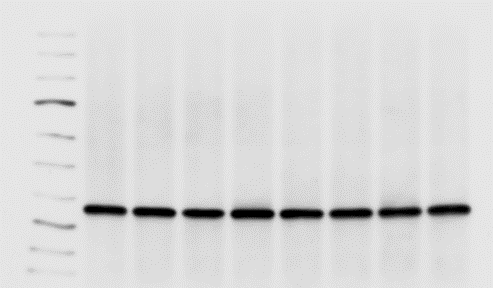
**

**GAPDH 36KD**

**E-cadherin 135KD**


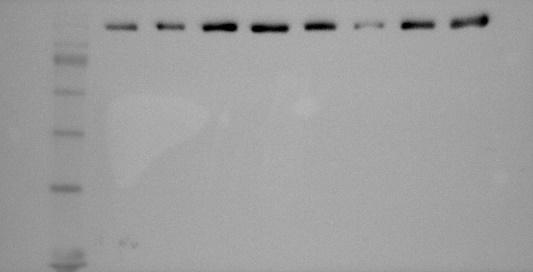

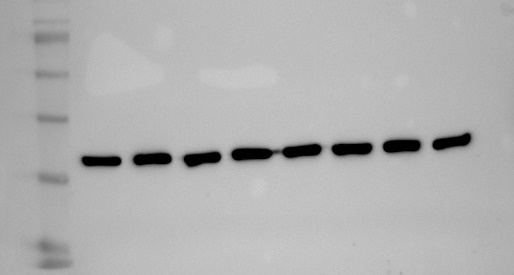


**N-cadehrin 130KD**

**GAPDH 36kD**


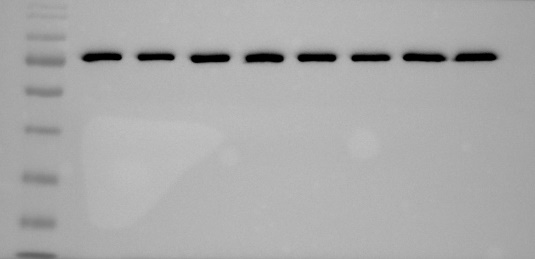

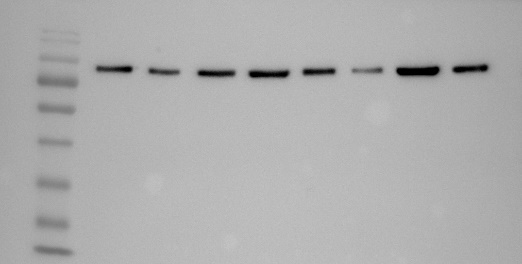


**p-PI3K 84KD**

**PI3K 83KD**


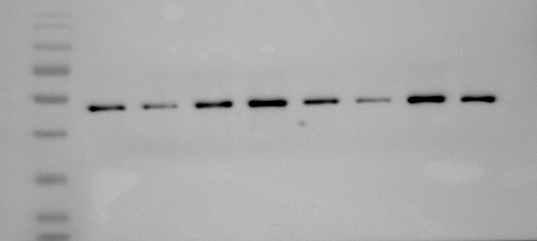
 **
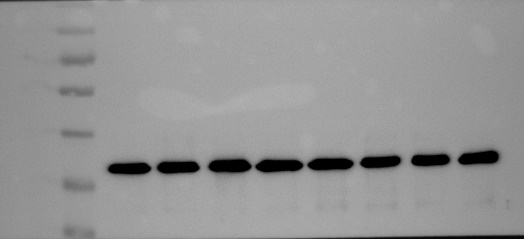
**

**GAPDH 36KD**

**Vimentin 54KD**

**
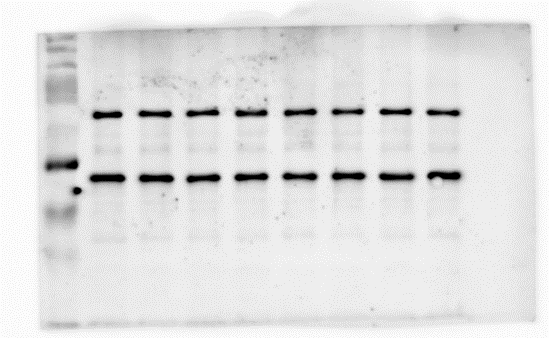

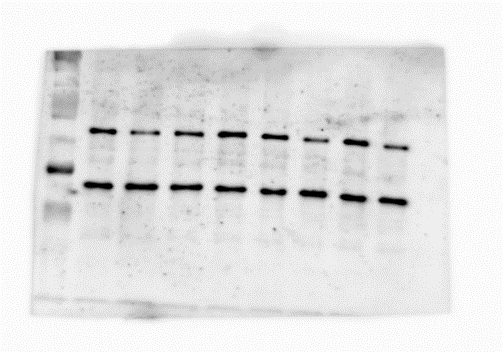
**

**p-AKT 55KD**

**AKT 56KD**

**GAPDH 36KD**

**GAPDH 36KD**

**
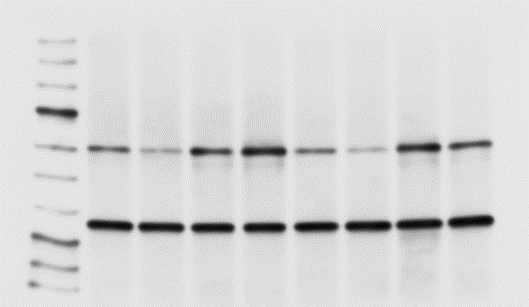
**

**GAPDH 36KD**

**PTBP3 60KD**

**Supplementary Figure 3 Original Western blotting images of Figure 6**

**
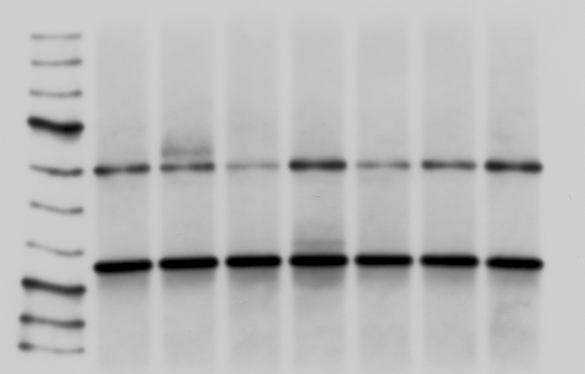

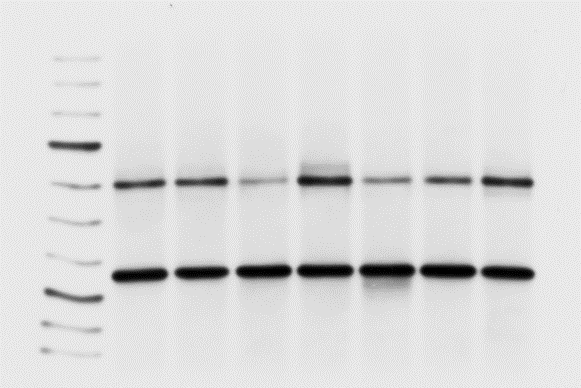
**

**PTBP3 60KD**

**GAPDH 36KD**

**PTBP3 60KD**

**GAPDH 36KD**

**
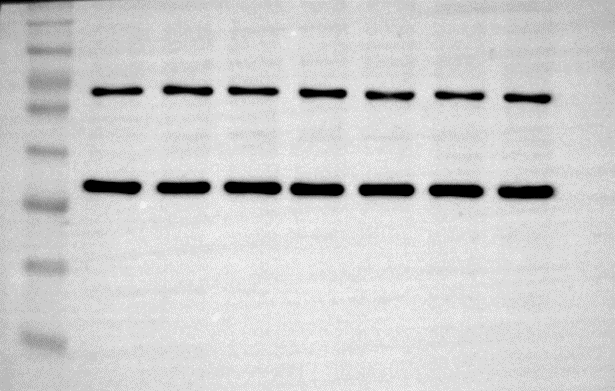

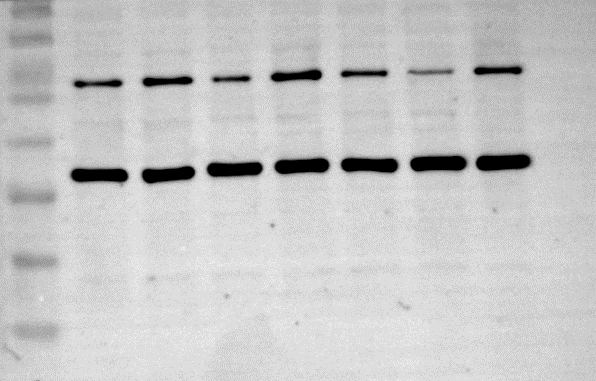
**

**GAPDH 36KD**

**P-AKT 55KD**

**GAPDH 36KD**

**AKT 56KD**

**
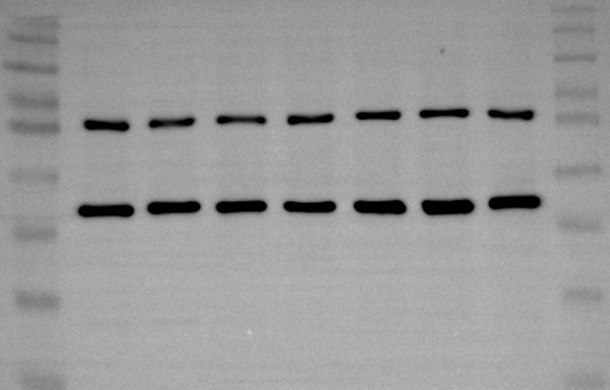

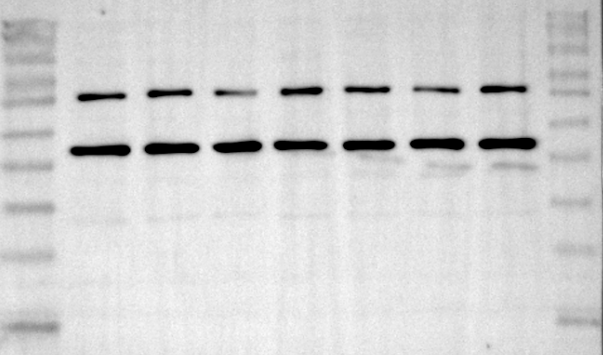
**

**GAPDH 36KD**

**P-AKT 55KD**

**GAPDH 36KD**

**AKT 56KD**

**
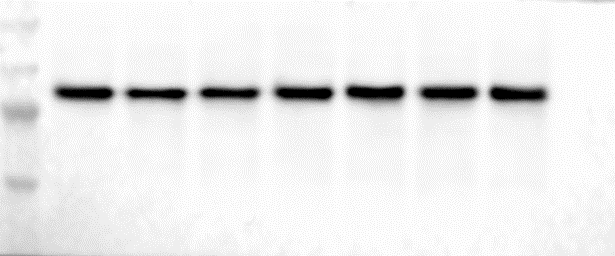

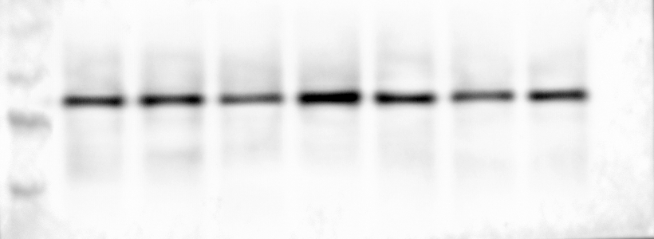
**

**p-PI3K 84KD**

**PI3K 83KD**

**
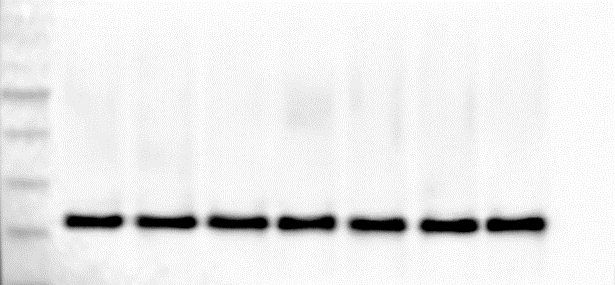

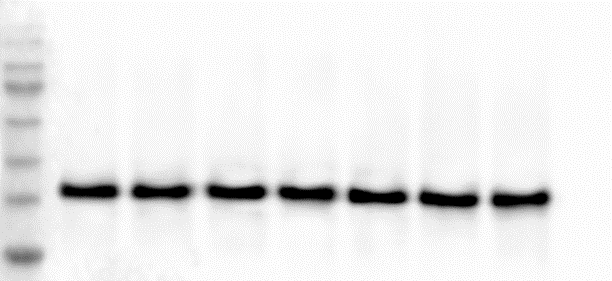
**

**GAPDH 36KD**

**GAPDH 36KD**

**
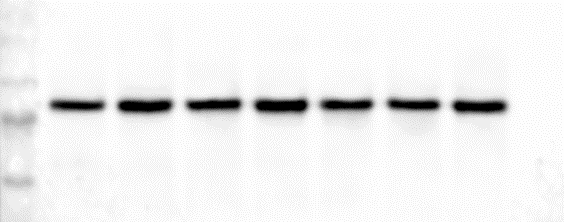

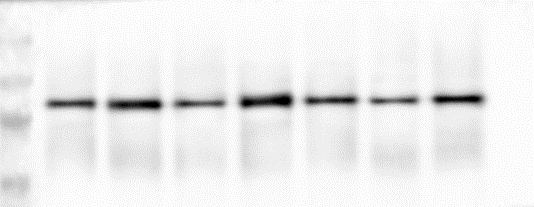
**

**PI3K 83KD**

**p-PI3K 84KD**

**
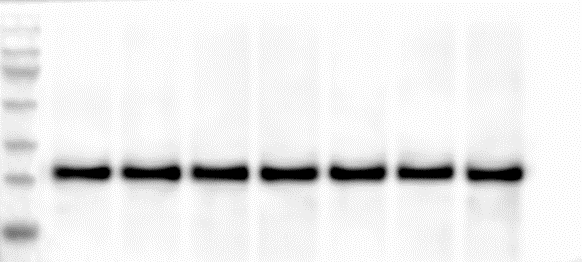

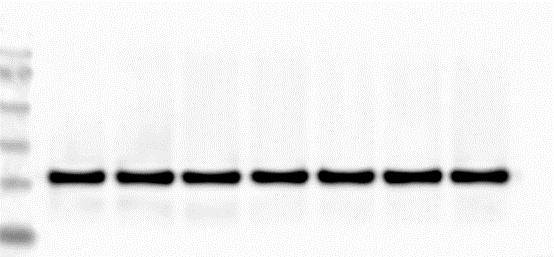
**

**GAPDH 36KD**

**GAPDH 36KD**
